# Research progress on the relationship between the TOR signaling pathway regulator, epigenetics, and tumor development

**DOI:** 10.3389/fgene.2022.1006936

**Published:** 2022-09-23

**Authors:** Jiaen Sun, Minglei Yang, Weidi Zhao, Fajiu Wang, Liangwei Yang, Chuntao Tan, Tianjun Hu, Huangkai Zhu, Guofang Zhao

**Affiliations:** ^1^ School of Medicine, Ningbo University, Ningbo, Zhejiang, China; ^2^ Department of Thoracic Surgery, Hwa Mei Hospital, University of Chinese Academy of Sciences, Ningbo, Zhejiang, China; ^3^ Department of Cardiac and Vascular Surgery, Hwa Mei Hospital, University of Chinese Academy of Sciences, Ningbo, Zhejiang, China

**Keywords:** TOR signaling pathway regulator, protein phosphatase, cancer, epigenetic, metabolism

## Abstract

Almost all cellular activities depend on protein folding, signaling complex assembly/disassembly, and epigenetic regulation. One of the most important regulatory mechanisms responsible for controlling these cellular processes is dynamic protein phosphorylation/dephosphorylation. Alterations in phosphorylation networks have major consequences in the form of disorders, including cancer. Many signaling cascades, including the target of rapamycin (TOR) signaling, are important participants in the cell cycle, and dysregulation in their phosphorylation/dephosphorylation status has been linked to malignancies. As a TOR signaling regulator, protein phosphatase 2A (PP2A) is responsible for most of the phosphatase activities inside the cells. On the other hand, TOR signaling pathway regulator (TIPRL) is an essential PP2A inhibitory protein. Many other physiological roles have also been suggested for TIPRL, such as modulation of TOR pathways, apoptosis, and cell proliferation. It is also reported that TIPRL was increased in various carcinomas, including non-small-cell lung carcinoma (NSCLC) and hepatocellular carcinomas (HCC). Considering the function of PP2A as a tumor suppressor and also the effect of the TIPRL/PP2A axis on apoptosis and proliferation of cancer cells, this review aims to provide a complete view of the role of TIPRL in cancer development in addition to describing TIPRL/PP2A axis and its epigenetic regulation.

## 1 Introduction

Cancer has overtaken cardiovascular disease as the leading cause of death in many countries. Based on cancer registry data, five frequent malignancies, including breast, prostate, lung, colorectal cancers, and hepatocellular carcinomas (HCC) account for more than half of the yearly observed mortality in the 2016–2020 period (Peto, 2001; Hiatt et al., 2022). Phosphorylation is one of the most well-researched post-translational modifications (PTMs), and it governs a range of biological processes such as cell differentiation, proliferation, death, and cell signaling in healthy conditions. Changes in phosphorylation networks, on the other hand, have major consequences in the form of disorders, including cancer. Many signaling cascades, such as the target of rapamycin (TOR) signaling, are important participants in the cell cycle, and dysregulation in their phosphorylation-dephosphorylation cycle has been linked to many malignancies (Hartl et al., 2011; Brautigan, 2013; Singh et al., 2017). The phosphorylation status of proteins, which controls their function, is determined by the competition between protein kinases and protein phosphatase (PP). Phosphoprotein phosphatases (PPPs) are a class of enzymes (PP1, PP2A, PP2B, and PP2C) necessary for most serine and threonine dephosphorylation in cells.

This is in contrast to the over 400 protein kinases accountable for serine and threonine phosphorylation (Manning et al., 2002). The formation of multimeric holoenzymes is how PPPs attain the specificity and selectivity required of their substrates. The assembly of PPP holoenzymes is subject to stringent regulation, and alterations in the cellular repertoire of PPPs have been connected to cancer development. Combined with the closely related phosphatase PP1, protein phosphatase 2A (PP2A) is responsible for more than 90 percent of the phosphatase activity that occurs within cells. The formation of various heterotrimeric holoenzymes is what controls the functions of PP2A, it is structurally comprised of a scaffold (PP2Aa), a regulatory (PP2Ab), and a catalytic (PP2Ac) subunit. The catalytic c subunit is also called PP2Ac. In addition, a variable B subunit that comes from four regulatory families, including B, B′, B″, and B‴ (Shi, 2009; Wu et al., 2017). Biogenesis of various PP2A holoenzymes involves PP2A-specific chaperones, α4 and PP2A phosphatase activator (PTPA), and methylation enzyme leucine carboxyl methyltransferase (LCMT)-1 (Guo et al., 2014).

It is essential for normal physiology to maintain stringent control over the cellular PP2A holoenzymes, and abnormalities in PP2A regulation have been linked to the development of many solid cancers as well as leukaemias (Remmerie and Janssens, 2019). For example, in the chronic myeloid leukaemia, the PP2A phosphatase activity was suppressed. An essential PP2A inhibitory protein is the TOR signaling pathway regulator (TIPRL) (McConnell et al., 2007). The TIPRL structure is a unique butterfly shape consisting of the main core of the antiparallel β-sheet (Scorsato et al., 2016). TIPRL, as a binding partner for mammalian α4/yeast type 2A-associated protein of 42 kDa (Tap42), is the mammalian ortholog of the yeast TAP42 interacting protein of 41 kDa (TIP41) (Jacinto et al., 2001). Ataxia telangiectasia mutated (ATM) and ATM and Rad3-dependent phosphorylation events (ATR) are suppressed by TIPRL interaction with PP2Ac, PP type 4 (PP4), or PP type 6 (PP6), together with α4, excluding A and B subunits, finally inhibiting PP function (Nanahoshi et al., 1998; Smetana and Zanchin, 2007a). The TOR signaling pathway is also under the control of the interaction between TIPRL and PP2A (Nakashima et al., 2013). In addition to this pathway, TIPRL has a crucial function in the DNA damage response, apoptosis, and cell proliferation. It is also reported to be increased in various carcinomas, which enables cancer cells to escape apoptotic processes (Song et al., 2012).

Considering the function of PP2A as a tumor suppressor and also the effect of the TIPRL/PP2A axis on apoptosis and proliferation of cancer cells, this review aimed to provide a complete view of the role of TIPRL in tumor development in addition to an overview of the TIPRL/PP2A axis and its epigenetic regulation.

## 2 Overview of TIPRL/PP2A axis

In most of the organisms, the regulation of cell division, cell growth, metabolism, and stress responses depend on the dynamic phosphorylation/dephosphorylation cycle. As one of the most prevalent mechanisms to regulate protein activity, alterations in phosphorylation status affect efficiency, stability, localization, as well as the protein-protein interactions. A phosphate group from ATP is transferred by eukaryotic protein kinases to the hydroxyl groups of serine, threonine, and tyrosine (Ser/Thr/Tyr) residues, while PPs hydrolyze the phosphoester linkage to release phosphate and dephosphorylating protein. In comparison to protein kinases, PPs have received a far smaller amount of research attention for various reasons. At one point, they were viewed as housekeeping (HK) enzymes without significant regulatory activities. Nevertheless, this view has long since altered, and it is now acknowledged that PPs are complexly regulated and extremely selective to various protein substrates (DeLong, 2006; Uhrig et al., 2013a; Brautigan, 2013). The four diverse gene families that make up eukaryotic PPs each have their unique active site signatures. These gene families are as follows: 1) Ser/Thr-specific PPPs, 2) Mg^2+^-dependent PPs, 3) Aspartic acid-based PPs, and 4) phospho-Tyr phosphatases (PTPs) (Kerk et al., 2008; Dennis and Bradshaw, 2011).

Approximately 80% of the PP activity in eukaryotic cells is accounted for by the Ser/Thr-specific PPPs, which is one of the most highly conserved proteins among eukaryotic species (Janssens and Goris, 2001). PP1, PP2B, PP4, PP5, PP6, and PP7 are the main subgroups of the PPP family (Uhrig et al., 2013b; Maselli et al., 2014; Rezaiemanesh et al., 2021). The ten catalytic subunits are responsible for the majority of the dephosphorylation of Ser and Thr in the cell. These subunits include PP1α, PP1β, PP1γ, PP2Acα, PP2Acβ, PP2Bc, PP4c, PP5c, PP6c, and PP7c. From yeast to humans, the C subunits of PP2A, PP4, and PP6 are closely associated and highly conserved (Chen et al., 2017).

**FIGURE 1:**
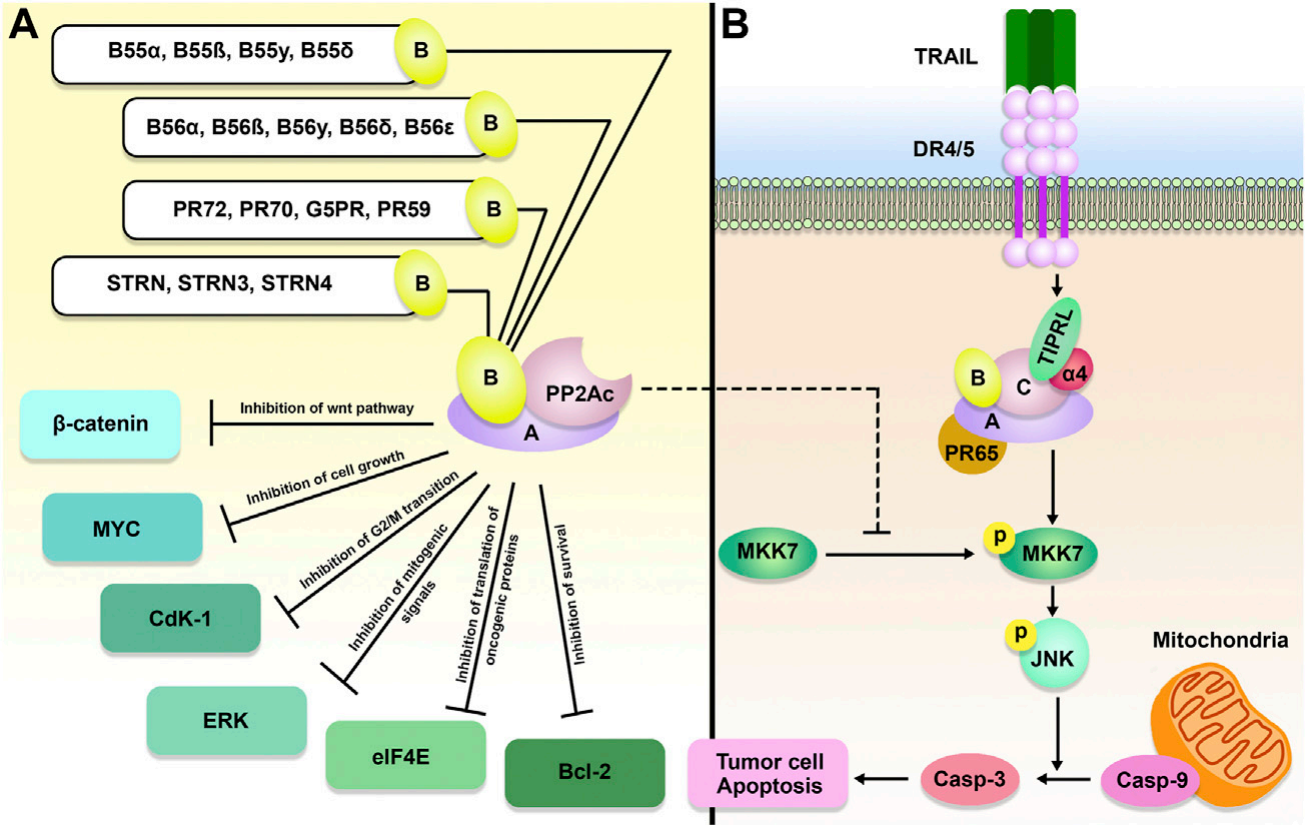
TIPRL/PP2A axis and TOR signaling pathway. Active PP2A holoenzyme is an important inhibitor of the TOR signaling pathway. Besides, PP2A inhibits cancer cell growth and survival through dephosphorylation of 4E-binding protein 1 4EBP-1) and S6 kinase (S6K). PP2A holoenzymes can be inhibited as a result of α4/TIPRL-mediated recycling and disassembly of scaffold **(A)** and regulatory **(B)** subunits.

During the process of looking for components of the TOR signaling pathway in yeast, PP2A was shown to be an essential player in the route (Figure 1). As mentioned earlier, subunits A, B, and C make up the PP2A holoenzyme (Xu et al., 2006). The A and C subunits each only have two unique isoforms, while the B subunit is responsible for the majority of PP2A’s functional variety. There are currently 16 recognized forms of the B subunit, and several incorporate further splice variants (Vainonen et al., 2021). TIPRL and Tap42/α4 could engage PP2Ac concurrently, generating a stable ternary complex, according to Smetana et al. (2007b). Tap42/α4, as a regulatory component of PP2A, is a downstream effector of the TOR protein kinase. TOR is a protein kinase that controls cell growth in yeast and mammals by coordinating it with the availability of nutrients and environmental factors. PP2A plays a critical part in TOR signaling through its interaction with phosphorylated Tap42/α4. Despite being initially discovered as a TAP42 binding protein, *S. cerevisiae* TIP41 operated antagonistically as a suppressor in the TOR axis (Jacinto et al., 2001). Within *S. cerevisiae*, TIP41 was found to engage with the PP2A, PP4, and PP6 C subunits (Gingras et al., 2005). Human TIPRL also binds directly to the PP2A, PP4, and PP6 C subunits. TIPRL, unlike yeast TIP41, can stimulate the activity of the TOR signaling pathway via its interaction with PP2Ac (Nakashima et al., 2013). Nakashima et al. showed that TIPRL inhibited dephosphorylation of TOR Complex 1 (TORC1) substrates after amino acid deprivation, but TIPRL silencing inhibited phosphorylation of those substrates following amino acid exposure. TIPRL was found to be linked with the PP2Ac, which was essential for TIPRL’s impact on TORC1 function (Nakashima et al., 2013).

PP2A, PP4, and PP6 all form distinct clusters within the PPPs, indicating a previously shared ancestor (Uhrig et al., 2013b). A YFL (tyrosine-phenylalanine-leucine) signature, which is conserved among them, can be found near the C-terminal end of the C subunit. PTMs of the catalytic subunits influence holoenzyme assembly by phosphorylating the Tyr residues and methylating the leucine (Leu) residues, respectively (Longin et al., 2007; Janssens et al., 2008). It has been reported that PP2A activity is reduced when phosphorylation occurs at the Tyr (Brautigan, 2013). It has been demonstrated that methylation of the Leu can affect the binding of regulatory subunits, and as a result, methylation is a component of the system that gives protein complexes their substrate specificity (Janssens et al., 2008).

Similar to α4, TIPRL is a conserved binding protein to all PP2A-family phosphatases, particularly PP2A, PP4, and PP6 (Gingras et al., 2005; Smetana and Zanchin, 2007a). The fact that all of the residues in PP2A participating in TIPRL binding are identical in PP4 and the fact that there is only one residue that is different in PP6 suggests that TIPRL interacts with PP4/PP6 in a manner that is analogous to how it interacts with PP2A. The methylation enzyme, α4, and TIPRL were among the regulatory proteins that PP4 and PP6 continuously shared with PP2A (McConnell et al., 2007; Jiang et al., 2013). In reaction to demethylation, TIPRL acts as a dynamic PP2A inactivator and inhibits the phosphatase’s active site of PP2A, resulting in PP2A disassembly. Besides, Scorsato et al. study suggested that TIPRL could strongly bind to the original PP2A-tail over its tyrosine-phosphorylated counterpart. (Scorsato et al., 2016). The mechanism for dynamic assembly/disassembly of PP2A holoenzymes is established by these findings, which also highlight a dynamic component of PP2A regulation. The main feedback loop for efficient control of PP2A holoenzyme turnover is provided by α4/TIPRL, which does not affect the cellular concentration of PP2Ac. These findings explain how PP2Ac concentrations stay stable throughout the cell cycle in mammalian cells (Virshup and Shenolikar, 2009). A key signaling switch governing the stability and disintegration of the holoenzyme is provided by the methylation of the PP2Ac tail in this feedback loop. Demethylation of the PP2Ac tail is essential for the successful attack by TIPRL on the PP2A active site (Rosales et al., 2015). Furthermore, during DNA damage or cellular stress, PP2A holoenzymes may be downregulated as a result of α4/TIPRL-mediated recycling. The activity of PP2A in DNA damage-induced ATM/ATR signaling was discovered to be suppressed by TIPRL (Kong et al., 2009).

## 3 Epigenetic regulation of the TIPRL/PP2A axis

To rapidly adjust gene expression in response to alterations in the cellular environment, a broad and complex network of cellular activities balance the reading, deposition, and elimination of epigenetic markers. The three primary types of these epigenetic modifiers are “readers,” “writers,” and “erasers.” On DNA and histones, epigenetic “writers” and “erasers” are responsible for the deposition and removal of epigenetic markers like methylation (Audia and Campbell, 2016; Hyun et al., 2017). The deposited marks are interpreted by the epigenetic readers, which then either engage transcriptional co-factors or further chromatin remodeling complexes to particular places on DNA. To maintain normal gene expression, these modifiers work together in a careful equilibrium. This equilibrium is disturbed by epigenetic dysregulation, which contributes to the aberrant stimulation of oncogenic signaling networks and the beginning stages of the tumorigenesis process (Vicente-Dueñas et al., 2018; Gil and Vagnarelli, 2019). Protein phosphorylation seems to substantially affect the epigenetic state of cancer cells, according to an increasing body of research conducted in recent years (Liu et al., 2016; Yang and Li, 2020). In response to various stimuli inside the cell, kinases and phosphatases make quick and reversible modifications to proteins. These modifications eventually result in a change in the function, localization, and interaction of partners. Anti-tumor treatments have mostly focused on kinase inhibitors, contributing to customized medicine’s growth (Dolgin, 2017). Phosphatases, on the other hand, play an important and sometimes underestimated function in inhibiting neoplastic signaling and are developing as potential targets for medicinal substances (Westermarck, 2018; Vainonen et al., 2021).

The composition of the PP2A holoenzyme is very important to both the function of PP2A and TIPRL and their respective contributions to the process of carcinogenesis. The extraordinary multi-branching mechanism offered by the butterfly-shaped TIPRL may establish some extremely integrative connections with PP2Ac and the A subunit. It is probable that complex interactions are responsible for explaining the multidimensional roles that TIPRL plays in regulating PP2A. These roles include inactivating the phosphatase active site, responding to demethylation, and suppressing some molecular interactions pertinent to holoenzyme formation. In a cellular environment, methylation acts as a protection mark for PP2A holoenzymes, allowing them to avoid being attacked by TIPRL (Vafai and Stock, 2002; Sontag et al., 2004). TIPRL can also be inhibited by single amino acid alterations such as I136T, D71L, D198N, and M196V according to results from reverse two-hybrid experiments (Smetana and Zanchin, 2007b).

Methylation and acetylation of epigenetic targets are facilitated by proteins that are regulated by PP2A. PP2A is responsible for dephosphorylating bromodomain-containing 4 (BRD4), which prevents BRD4 from attaching to acetylated residues and speeds up the transcription process. As a transcriptional and epigenetic regulator that plays an important role in cancer development, BRD4 accumulates at euchromatic sites to stimulate gene expression (Donati et al., 2018). BRD4 is shown to be more likely to be associated with activating acetylation marks after being phosphorylated by casein kinase II (CK2), making BRD4 hyperphosphorylation a predictor of poor prognosis in several malignancies (Wu et al., 2013; Sanz-Álvarez et al., 2021). Additionally, as shown by ERK phosphorylation and increased kinase activity, downregulation of CK2 can result in suppression of the mammalian target of rapamycin (mTOR) cascade, downregulation of Raptor expression levels, and stimulation of the extracellular signaling-regulated protein kinase 1/2 (ERK1/2) signaling mechanism (Olsen et al., 2012).

Histone deacetylase (HDAC) 4/5/7 is dephosphorylated by PP2A, which also prevents HDAC from binding to 14-3-3. This allows HDAC to become nuclear-localized, leading to histone deacetylation. It has been demonstrated that the differentiation status of fibroblasts can be affected by HDAC4 regulation by PP2A (Li et al., 2017). Considering fibroblast infiltration and stimulation contributing to metastasis and treatment resistance in some cancers, fibroblast reprogramming by tumor cells has been established as a crucial aspect of tumor biology (Underwood et al., 2015; Butti et al., 2021; Steele et al., 2021). In addition, HDAC8 overexpression increased the amount of apoptosis generated by cisplatin in H1299 pulmonary cancer cells by suppressing the expression of TIPRL. Additionally, the knockdown of TIPRL increased the amount of apoptosis that was induced in cisplatin-treated cells. In a way dependent on HDAC8, the isoproterenol administration also reduced the amount of transcription of the TIPRL gene caused by cisplatin (Park and Juhnn, 2017). TIPRL, as mentioned above, is a negative regulator of PP4. Rosales et al. study showed that the TIPRL depletion boosted PP4 phosphatase activity and the assembly of the functional PP4 complex, which is known to dephosphorylate H2A histone family member X (H2AX). TIPRL upregulation enhanced H2AX phosphorylation in response to DNA damage, whereas TIPRL silencing reduced H2AX phosphorylation. TIPRL upregulation also caused cell death in regard to stress, whereas TIPRL suppression shields cells from genotoxic chemicals, in association with H2AX (Rosales et al., 2015).

A low level of PRMT1/5 methylase activity at the H4R3me2 methylation site is connected with high levels of PP2A activity. Cancer determines the effect of this regulation on gene expression. The most common SDMA methyltransferase is PRMT5, which is linked to gene repression (Yang and Bedford, 2013). There is a mountain of evidence pointing to PRMT5 possessing an oncogenic function in various malignancies, including prostate, pancreatic, and colorectal cancers (Lee et al., 2021; Beketova et al., 2022).

PP2A is responsible for dephosphorylating ten-eleven translocation (TET)-2, which decreases the stability of TET2. This activity inhibits the elimination of methylcytosine in an effective manner. The TET proteins play a key role in the successive oxidation of 5′-methylcytosine to restore the cytosine base’s unaltered state and serve primarily as tumor suppressors (Wu and Zhang, 2017). MYC is frequently expressed constitutively in cancerous tissue. There is a correlation between the dysregulation of the MYC family oncogene and a poor prognosis in patients. This dysregulation occurs in more than fifty percent of human malignancies. Researchers have shown a correlation between MYC upregulation and aggressive human prostate cancer, triple-negative breast cancer, and many different human cancers (Gurel et al., 2008; Palaskas et al., 2011; Dang, 2012). PP2A affects the stability of MYC protein in a direct and immediate post-translational manner, in addition to its potential to regulate MYC transcription (Arnold and Sears, 2006). The activity of PP2A is frequently suppressed during malignancy, which results in an increase in the phosphorylation and the transcriptional activity of MYC (Westermarck and Neel, 2020). The complex processes by which the TIPRL/PP2A axis affects the epigenetic status of cancer cells are summarized in Table 1.

**TABLE 1:** Epigenetic targets of TIPRL/PP2A axis.

Target	Effect	Reference
H3	PP2A reduced the MYC and MYC-related gene transcription	Huang et al., (2005); Komar and Juszczynski, (2020)
BRD4	PP2A decreased BRD4-associated gene transcription	Sanz-Álvarez et al. (2021)
HDAC4	PP2A inhibited the HDAC binding to 14-3-3	Paroni et al., (2008); Li et al., (2017)
HDAC7	PP2A inhibited the HDAC binding to 14-3-3	Martin et al. (2008)
HDAC8	TIPRL is negatively associated with increased apoptosis	Park and Juhnn, (2017)
PRMT1	PP2A inhibited PRMT1 activity	Ichikawa et al. (2020)
H2AX	TIPRL upregulation enhanced H2AX phosphorylation in response to DNA damage	Chowdhury et al., (2005); Rosales et al., (2015)
	PP2A promoted DNA damage resolution	
TET2	PP2A decreased the stability of TET2	Kundu et al. (2020)

## 4 The role of the TIPRL/PP2A axis in cancer development

**FIGURE 2:**
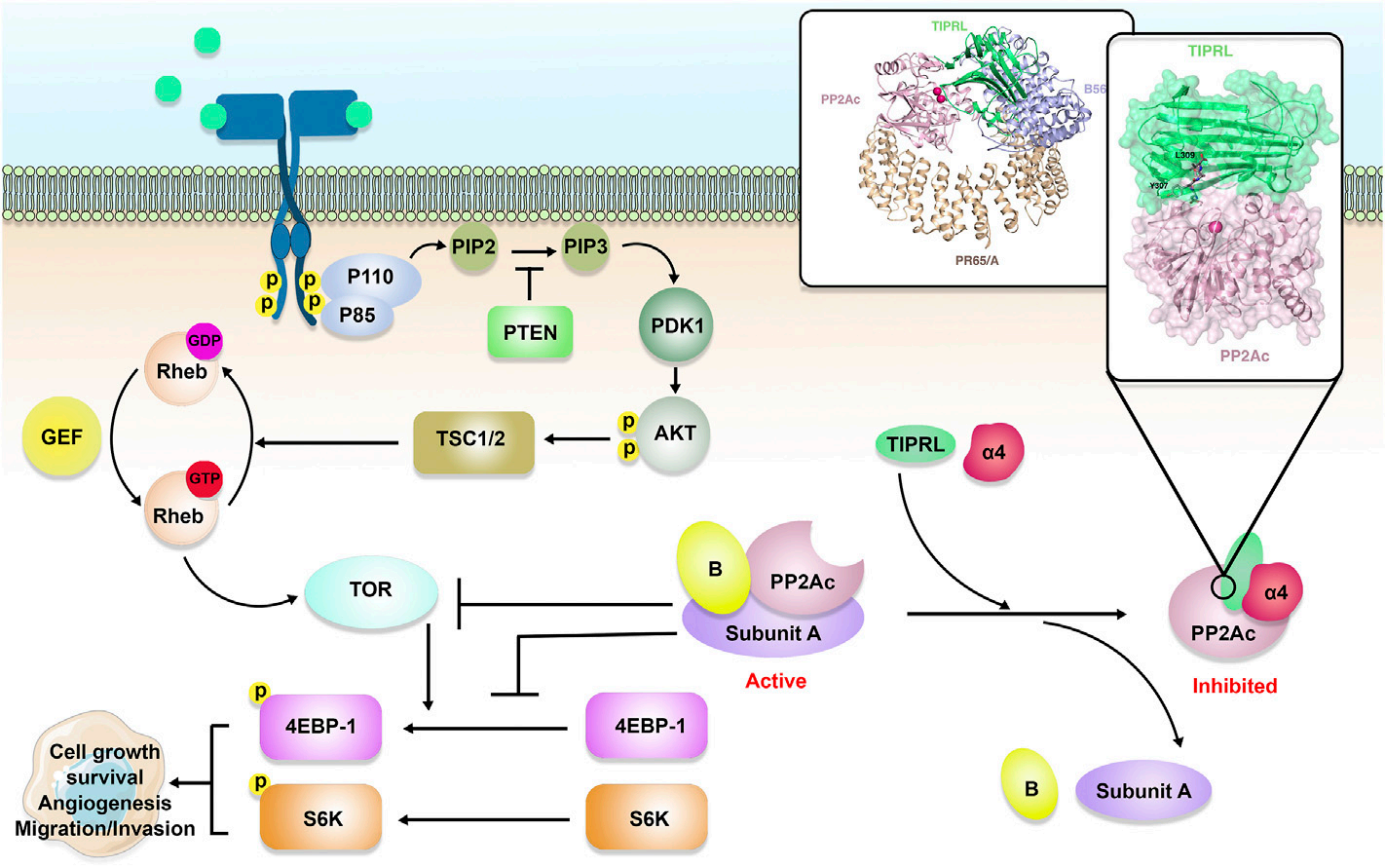
The role of the TIPRL/PP2A axis in cancer development. PP2A is comprised of a scaffold, a regulatory, and a catalytic (C or PP2Ac) subunit, a variable B subunit that comes from four major families, including B, B′, B″, and B‴. PP2A holoenzyme, as a tumor suppressor, can inhibit MYC, Cyclin-dependent kinase 1 (Cdk1), B-cell lymphoma 2 (Bcl-2), Extracellular signal-regulated kinase (ERK), wnt/β-catenin, and eukaryotic translation initiation factor 4E (eIF4E). In addition to inhibition of PP2A, TIPRL can also participate in TRAIL-induced apoptosis and subsequent phosphorylation of MAP kinase 7 (MKK-7) and c-Jun N-terminal kinase (JNK), resulting in cancer cell apoptosis.

During tumorigenesis, PP2A dysregulation can profoundly impact gene expression, genomic stability, and PTM. Because it inhibits the activity of a wide variety of well-known oncogenes, such as MYC, BCL-2, ERK, and AKT, the PP2A holoenzyme is considered a tumor suppressor (Lin et al., 2006; Ruvolo et al., 2011) (Figure 2). In accordance with this function, the PP2A activity is frequently inhibited during cancer (Ruvolo, 2016). PPP2R1A, also known as the PP2A Aα scaffolding subunit, possesses the greatest mutation rate out of all the PP2A subunits in ∼1% of all malignancies (Sangodkar et al., 2016). It has been demonstrated that these mutations promote transformation and tumor growth by impairing B or C subunit binding, suppressing the global activity of PP2A (Chen et al., 2005; Haesen et al., 2016).

TIPRL expression pattern in non-small-cell lung carcinoma (NSCLC) was studied by Xu et al. (2020) utilizing The Cancer Genome Atlas (TCGA) dataset. TIPRL was shown to be elevated in NSCLC and was related to an aggressive metastasis stage. TIPRL overexpression was correlated with significantly shorter survival in NSCLC patients. TIPRL silencing inhibited A549, a human Caucasian lung carcinoma cell line, invasion. Besides, TIPRL knockdown dramatically increased cadherin transcription while suppressing vimentin transcription in A549 cells. Another study demonstrated that TIPRL and has-circ-0010235 were upregulated in NSCLC tissues. In NSCLC cells, has-circ-0010235 knockdown or increase of miR-433-3p boosted apoptosis while suppressing proliferation and autophagy. Has-circ-0010235 sponged miR-433-3p to increase TIPRL expression and hence influence NSCLC development. *In vivo*, has-circ-0010235 depletion also inhibited tumor development (Zhang et al., 2021).

According to Jun et al., the expression of TIPRL in liver cancer tissues is highly associated with levels of LC3, a central adaptor in the autophagy cascade, and CD133, also known as prominin-1 and AC133. TIPRL, CD133, and LC3 were shown to be significantly upregulated in hepatocellular carcinomas (HCCs) in comparison to the surrounding normal tissues. TIPRL was found to have a major impact on HCC patient’s prognosis. TIPRL knockdown dramatically decreased LC3 and CD133 gene expression, as well as cell survival of HCC cell lines, which were increased by aberrant TIPRL level (Jun et al., 2019). Another research by Jun et al. found that TIPRL and LC3 were significantly upregulated in adult hepatocyte-derived liver disease, but they were downregulated in intrahepatic carcinomas (iCCA). The TIPRL transcription has been found to be the most accurate predictor of survival in patients suffering from liver cancer. This evidence suggests that TIPRL promoted liver cancer cell proliferation by inducing cell survival. TIPRL, LC3, and CD133 had good efficacy in diagnosing patients with grade 1 iCCA, while TIPRL/LC3/CD133/CD44 axis demonstrated prognostication of both grade 1 HCCs and iCCA (Jun et al., 2021).

Through mRNA microarray analyses, Luan et al. suggested TIPRL as a new metastasis blocker in gastric cancer (GC). TIPRL expression was shown to be lower in clinical GC tissues, and it was linked to a higher metastasis, tumor stage, and a worse clinical prognosis. TIPRL has also been reported to be a direct target of miR-383-5p and miR-216a-5p. TIPRL Upregulation in GC cell lines decreased invasive characteristics, whereas TIPRL-deficient models had the opposite impact. TIPRL also promoted AMP-activated protein kinase (AMPK) activation, which in turn reduced phosphorylation of mTOR, 4EBP-1, and S6K, resulting in mTOR signaling deactivation and consequent inhibition of tumor invasion in GC (Luan et al., 2020).

Tumor necrosis factor (TNF)-related apoptosis-inducing ligand (TRAIL) is a potential anti-tumor drug (Figure 2), however, some cancerous cells are insensitive to it. Yoon et al. study showed that the co-treatment of Huh7 cells, a permanent HCC cell line, with TRT-0173 or TRT-0029, as TRAIL sensitizers, resulted in TRAIL-induced apoptosis because of suppression of the TIPRL-MKK-7 interaction and subsequent phosphorylation of MAP kinase 7 (MKK-7) and c-Jun N-terminal kinase (JNK). *In vivo*, these chemicals were injected into HCC xenograft tumors, resulting in inhibition of tumor development (Yoon et al., 2017). Yoon et al. also suggested that combining TRAIL with Taraxacum officinale F.H. Wigg (TO) treatment of Huh7 cells resulted in TRAIL-induced apoptosis through a similar pathway (Yoon et al., 2016). Tussilago farfara L. (TF) was proposed by Lee et al. (2014) as another TRAIL sensitizer. The combination of TF and TRAIL triggered apoptosis in Huh7 cells by inhibiting the TIPRL-MKK-7 interaction and increasing MKK-7 and JNK phosphorylation. According to Song et al. (2012) study, levels of TIPRL were higher in HCC tissues and cell lines in comparison to the non-tumor tissues. Silencing of TIPRL led to increased apoptosis. Treatment of HCC cells with small interfering RNA (siRNA) against TIPRL (siTIPRL) and TRAIL caused phosphorylation of MKK-7 and JNK and led to apoptosis, indicated by cleavage of caspase-8 and caspase-3. *In vivo*, injection of HCC xenograft tumors with siTIPRL and TRAIL led to the induction of tumor apoptosis.

## 5 Conclusion and future perspective

TIPRL is a novel PP2A inhibitor. It seems that demethylation of the PP2Ac tail is essential for TIPRL-mediated inhibition of PP2A. TIPRL/PP2A also control the methylation, acetylation, and phosphorylation of several epigenetic targets that are important in gene expression and cell cycle. In addition to the effects associated with PP2A inhibition, TIPRL has important regulatory effects in the TOR signaling pathway. Since PP2A holoenzyme is known as a tumor suppressor, its inhibition is expected to contribute to tumor progression. However, studies have shown that this theory is not entirely true. Indeed, there are studies indicating that the high expression of TIPRL is not always associated with tumor development and worse prognosis. Also, there are still contradictions regarding the relationship of TIPRL with the induction of apoptosis in cancer cells. Although TIPRL is expected to prevent cancer cell apoptosis by inhibiting PP2A, which is an inhibitor of BCL-2, a series of studies have shown that this protein can induce TRAIL-related cell death through the phosphorylation of MKK-7. This paradox in the results of studies can be caused by the endless complexities of intracellular signaling pathways and regulatory systems related to each other. However, to understand more about the function of TIPRL in cancer and to use it to enhance the anti-cancer effects of PP2A, it is necessary to conduct more clinical studies focusing on the epigenetic regulation of the TIPRL/PP2A pathway.
